# NEPHRO–ZEBRA-acute troponin increase in a kidney transplant recipient–the unknown knowns?

**DOI:** 10.1007/s40620-021-00990-7

**Published:** 2021-03-24

**Authors:** Nina Goerlich, Karin Klingel, Karl Stangl, Jens Gaedeke, Kai-Uwe Eckardt, Ralph Kettritz

**Affiliations:** 1 Department of Nephrology and Medical Intensive Care, Charité - Universitätsmedizin Berlin, Berlin, Germany; 2 Institute for Pathology and Neuropathology, University Tuebingen, Tuebingen, Germany; 3 Department of Cardiology and Angiology, Charité - Universitätsmedizin Berlin, Berlin, Germany; 4 Experimental and Clinical Research Center, Max Delbrueck Center for Molecular Medicine in the Helmholtz Association, Charité - Universitätsmedizin Berlin, Berlin, Germany

**Keywords:** Amyloidosis, Myocarditis, Cardiovascular disease, Cytomegalovirus, Kidney transplantation

## Case description

A 64-year-old kidney transplant recipient developed sudden, profound, muscle weakness in all extremities during a business trip to Berlin. On examination, the patient was awake, oriented, had normal cranial nerve function, and no sensory defects. His past medical history revealed focal-segmental glomerulosclerosis at the age of 5 years and kidney transplantations in 1988 and 2007. He had been on hemodialysis for two years and had had mild hypertension for more than 30 years. He received standard immunosuppression (methylprednisolone, mycophenolate mofetil, and Cyclosporin A (CyA)) complemented by simvastatin and an ACE inhibitor for mild hypertension.

## Labs:

Creatinine 3.3 mg/dL (Baseline 1.7 mg/dL).

CK 77,000 U/L (with < 2% MB).

Myoglobin 23,700 µg/mL.

Troponin T (high-sensitive) 136 ng/L.

GOT 290 U/L

GPT 109 U/L

Myositis was presumed and 3 g methylprednisolone was administered. A quadriceps biopsy showed muscle cell necrosis without inflammation. An autoimmune “myositis panel” (including ANA, dsDNA, ENA, and anti-synthetase antibodies) returned negative. Because combined CyA and statin treatment can cause rhabdomyolysis, both were discontinued. Skeletal muscle enzymes normalized over three weeks. At this time Troponin T increased to > 10,000 ng/L with only slightly elevated CK (237 U/L, 23% MB) and normal GOT and GPT. The patient did not have angina or ST-elevations in his electrocardiogram. The patient’s electrocardiogram did not show angina or ST-elevations. Echocardiography revealed an 18 mm septum without regional wall kinetic abnormalities and preserved ejection fraction. A heart muscle biopsy was performed.

### What is the cause of this patient’s tremendous troponinemia? What are your diagnostic tests to establish a diagnosis?

## Case solution

The patient recovered from rhabdomyolysis only to subsequently develop cardiac muscle lysis. Establishing the underlying cause for the skeletal myocyte damage was rather straightforward. Inflammatory myositis was ruled out in the muscle biopsy and toxic rhabdomyolysis was assumed, most likely from interactions between CyA and the statin. In contrast, elucidating the cause of the massive troponinemia indicating cardiac muscle breakdown was challenging but also educating education.

We excluded coronary artery occlusion via cardiac catheterization and considered the possibility of storage disease given the 18 mm interventricular septum with only mild hypertension. Endomyocardial biopsies established lymphocytic myocarditis, and a nested PCR detected human CMV DNA in the biopsy material confirmed by direct sequencing. Subsequently, we amplified CMV DNA from plasma, and detected CMV IgM and IgG antibodies indicating CMV reactivation or reinfection. With current prophylactic and preemptive antiviral treatment strategies, CMV disease occurs in approximately 15–30% of kidney allograft recipients at high risk within the first year after transplantation. Our patient had an unusual disease course in that CMV disease occurred 13 years after his second transplantation with a rather uncommon organ manifestation, namely myocarditis. A recent review described only 7 CMV myocarditis cases after solid-organ transplantation [1]. High-dose steroids administered shortly after admission possibly contributed to CMV reactivation in our patient. Could myocarditis explain such a high troponin T level? *Lauer *et al*.* found that Troponin T was more sensitive than CK and CK-MB in myocarditis [2]. The highest Troponin T level in their series was 8820 ng/L, somewhat lower than in our patient.

**Fig. 1:**
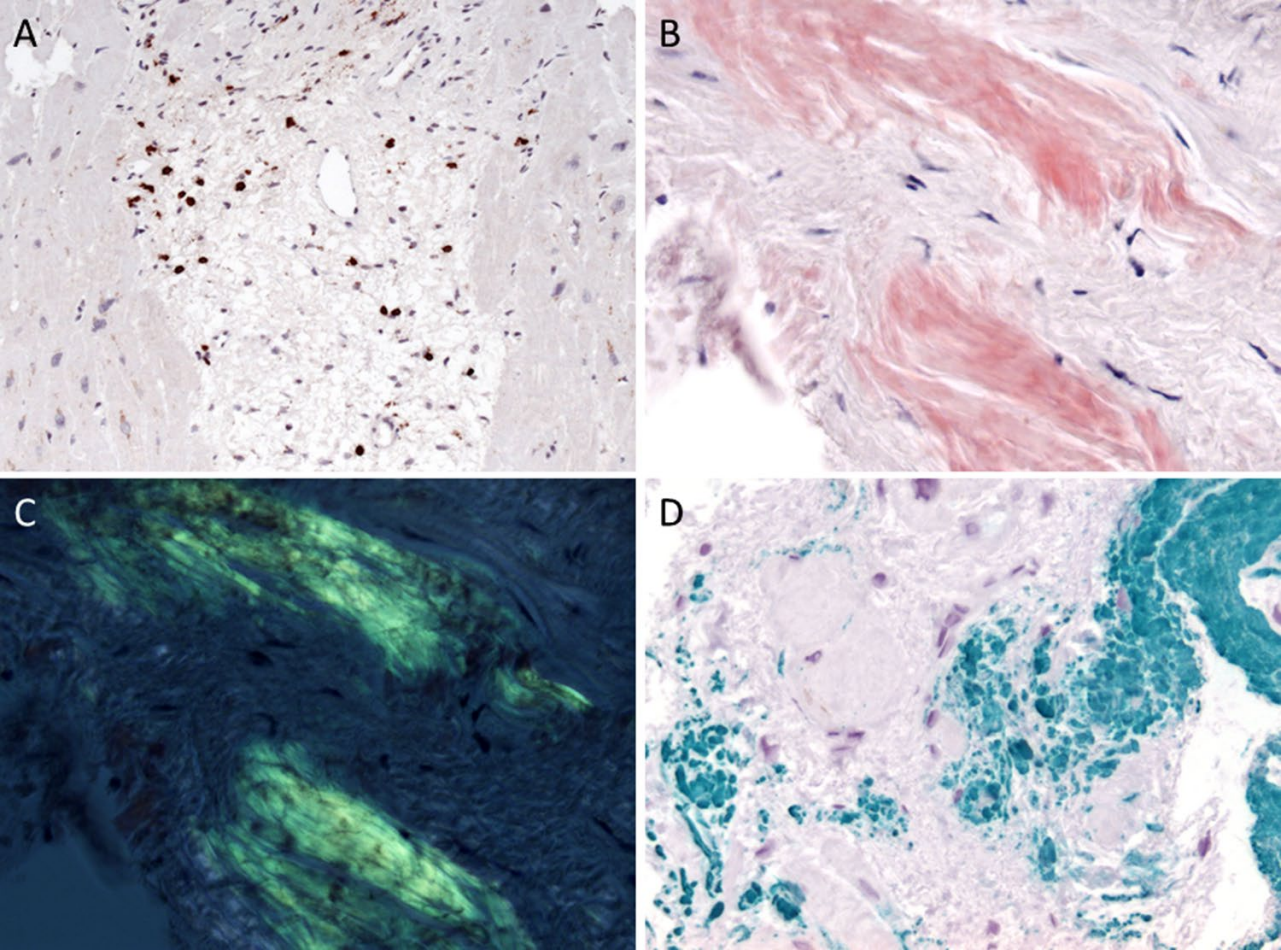
Endomyocardial biopsies. (**a**) Lymphocytic inflammation with numerous CD3^+^ T cells (× 200), (**b**) myocardial deposition of Congo-red material with (**c**) typical birefringence by polarized microscopy, (**d**) and serum amyloid A reactivity by immunohistochemical stainings (× 400)

Our second major histological finding was a strong interstitial and vascular deposition of amyloid in the myocardium with typical birefringence (Fig. 1) and strong reactivity for serum amyloid A (SAA) that possibly explains the discrepancy between mild hypertension and the markedly increased septum thickness. Nevertheless, why would this patient have de novo cardiac SAA? Only 11 of the 5112 amyloidosis patients from a large database developed de novo AA amyloidosis *post transplantation*, nine of whom were kidney transplant recipients [3]. Six of the eleven patients had renal, and none had cardiac amyloidosis. Did cardiac amyloidosis contribute to the tremendous Troponin T level in our patient? Cardiac AL amyloidosis caused increased Troponin T levels in two-thirds of the patients, but the reported levels were only mildly elevated [4]. Conceivably, the cardiac amyloidosis was synergistic with the myocarditis explaining these extremely high Troponin *T* values in our patient.

Our patient had none of the inflammatory diseases commonly associated with AA amyloidosis [5]. The authors of this review acknowledge that an idiopathic rest group remains and suggest that *SAA1.1* genotyping will eventually reveal common characteristics in this group. Our patient indeed carried a homozygous SAA1α gene mutation that is associated with a 3–sevenfold higher AA amyloidosis risk in Caucasians [6, 7]. Conceivably, his AA amyloidosis resulted from chronic inflammation that frequently occurs in immunosuppressed transplant patients together with his genetic predisposition. His persistently increased SAA protein and CRP levels support this assumption.

Finally, we suggest that SAA contributed to the extensive muscle wasting in our patient that made eGFR determinations impossible. Skeletal muscles express SAA1 receptors and binding of SAA1 activates NF-κB in myocytes [8]. NF-κB in turn mediates muscle atrophy and expression of the atrogenes *Trim63/MuRF1*, *Fbxo32/Atrogin1*, and *Fbxo30/MuSA1*. Such an inflammatory SAA-driven molecular mechanism possibly contributed to our patient’s rapid muscle atrophy.

### Follow-up

The patient improved after Valganciclovir was initiated. CMV copy number and troponin T declined slowly. He was able to leave the hospital on a walker.

## Conclusions

Our patient highlights the importance of toxic drug interactions, inflammatory disorders, and genetic modifiers, all encountered in solid-organ transplantation.

## References

[1] Scherger S, Mathur S, Bajrovic V (2020). CMV myocarditis in solid organ transplant recipients: a case series and review of literature. Transpl Infect Dis.

[2] Lauer B, Niederau C, Kuhl U (1997). Cardiac troponin T in patients with clinically suspected myocarditis. J Am Coll Cardiol.

[3] Sharpley FA, Fontana M, Gilbertson JA (2020). Amyloidosis diagnosed in solid organ transplant recipients. Transplantation.

[4] Dispenzieri A, Kyle RA, Gertz MA (2003). Survival in patients with primary systemic amyloidosis and raised serum cardiac troponins. Lancet.

[5] Brunger AF, Nienhuis HLA, Bijzet J, Hazenberg BPC (2020). Causes of AA amyloidosis: a systematic review. Amyloid.

[6] Cazeneuve C, Ajrapetyan H, Papin S (2000). Identification of MEFV-independent modifying genetic factors for familial Mediterranean fever. Am J Hum Genet.

[7] Gershoni-Baruch R, Brik R, Zacks N, Shinawi M, Lidar M, Livneh A (2003). The contribution of genotypes at the MEFV and SAA1 loci to amyloidosis and disease severity in patients with familial mediterranean fever. Arthritis Rheum.

[8] Hahn A, Kny M, Pablo-Tortola C (2020). Serum amyloid A1 mediates myotube atrophy via Toll-like receptors. J Cachexia Sarcopenia Muscle.

